# Prognostic value of three clinical nutrition scoring system (NRI, PNI, and CONUT) in elderly patients with prostate cancer

**DOI:** 10.3389/fnut.2024.1436063

**Published:** 2024-10-01

**Authors:** Shu-ying Li, Li-lin Wan, Yi-fan Liu, Yu-Wei Li, Xiang Huang, Rui-ji Liu

**Affiliations:** ^1^Sichuan Cancer Hospital and Institute, Sichuan Cancer Center, Cancer Hospital Affiliate to School of Medicine, University of Electronic Science and Technology of China, Chengdu, China; ^2^Department of Urology, Affiliated Zhongda Hospital of Southeast University, Nanjing, China; ^3^School of Medicine, University of Electronic Science and Technology of China, Chengdu, Sichuan, China; ^4^Department of Urology, Sichuan Provincial People's Hospital, School of Medicine, University of Electronic Science and Technology of China, Chengdu, China

**Keywords:** prostate cancer, malnutrition, prognosis, NRI, PNI, CONUT

## Abstract

**Background:**

Most of patients with prostate cancer (PCa) are elderly and have a long course of disease. Preoperative assessment of the patient's clinical nutritional status facilitates early intervention and improves patient prognosis.

**Methods:**

We assessed the nutritional status of PCa patients utilizing the Nutritional Risk Index (NRI), Prognostic Nutritional Index (PNI), and Controlling Nutritional Status (CONUT) scoring systems. Survival comparisons between groups were conducted using Kaplan-Meier curve analysis and log-rank tests, while Cox proportional hazards regression analysis was employed to identify independent prognostic factors. Furthermore, we implemented bootstrap-based optimism correction methods to validate the scoring systems and applied decision curve analysis to evaluate the non-inferiority of these three clinical nutrition scoring systems relative to the conventional American Joint Committee on Cancer (AJCC) staging.

**Results:**

In this study, malnutrition was diagnosed in 31.51% of the patients using the NRI, 13.02% using the PNI, and 88.28% using the CONUT score. After adjusting for confounders, normal nutritional status as defined by NRI and PNI emerged as an independent prognostic factor for prostate-specific antigen progression-free survival (PSA-PFS). However, nutritional status assessed by CONUT inaccurately predicted PSA-PFS. Normal nutritional status, as determined by all three scoring systems, was found to be an independent prognostic factor for progression-free survival (PFS). Following adjustments for optimistic estimates, the C-index for NRI in predicting both PSA-PFS and PFS remained the highest among the three scoring systems. The results of the DCA indicated that the C-index of all three scoring systems was higher than that of AJCC stage.

**Conclusions:**

NRI, PNI, and CONUT are convenient and clinically applicable scoring systems. A clinical malnutrition intervention may improve the prognosis of prostate cancer patients.

## Introduction

In 2021, there were about 720,000 new cases of tumors in men in the United States, of which prostate cancer (PCa) accounted for about 248,000 (22%), ranking first among all cancers. Further, PCa is the second leading cancer killer in men (1). In China, the incidence and mortality rate of prostate cancer is also increasing year by year (2). Recent years, despite meaningful advances in determining disease risk and identifying treatment options had been made, prostate cancer remains a long-term health risk for men.

At the time of diagnosis, most prostate cancer patients are elderly and have other chronic conditions such as hypertension, diabetes, chronic respiratory diseases, and neurological disorders. This not only affects the choice and timing of surgery, but also brings concerns about nutritional status and prognosis (3–5). It has been established that malnutrition is an important prognostic factor for cancer patients (6–8). Low body weight and cachexia reduce patients' tolerance to cancer treatment and therefore bring poor prognosis (9). Moreover, some laboratory indicators such as lymphocyte count, serum albumin and serum cholesterol reflect the patient's immune response and nutritional reserve during the treatment (10–12). In contrast to other clinical variables, these nutrient-related laboratory indicators are easy for clinicians to monitor and modulate. There is a lack of recognition, assessment, and active management of malnutrition in cancer patients. Most prostate cancer patients are elderly and have a long course of disease, meaning they are potentially at high risk of malnutrition.

Controlling Nutritional Status (CONUT) score, the Nutritional Risk Index (NRI), and the Prognostic Nutritional Index (PNI) is a nutritional assessment tool based on easily accessible laboratory indicators. Its superior prognostic value has been demonstrated in a clinical study of more than 5,000 acute coronary syndrome patients (13). Kuroda et al. performed a retrospective nutritional assessment using the CONUT score in 416 patients with curative resection of gastric cancer. They found that CONUT was not only a valid nutritional assessment tool, but also a predictor of long-term overall survival in gastric cancer patients (14). Okadome et al. used PNI scores in a prognostic study of patients with esophageal cancer (EC) and found that PNI is a prognostic biomarker in patients with EC (15). In addition, a study of postoperative complications and prognosis of patients with colorectal cancer showed that patients with low NRI ( ≤ 98) had higher incidence of postoperative complications and poorer prognosis (16). However, the relationship between nutritional status and prognosis in men with prostate cancer patients is still unknown.

In this study, we applied these three nutritional scoring systems to assess the clinical nutritional status and explore their prognostic value in prostate cancer patients.

## Patients and methods

### Study population

This is a retrospective study that included 384 prostate cancer patients diagnosed between January 2017 and January 2024 at our center. Histological confirmation of prostate adenocarcinoma is obtained by prostate biopsy or transurethral resection. We excluded patients with other primary tumors, active infections, and immunological disorders. All patients were hospitalized and treated systematically with completed data on height, weight, serum albumin levels, peripheral blood lymphocyte counts, total cholesterol concentration, and serum PSA. The ethics committee waived informed patient consent because of the anonymity of the data and the retrospective nature of the study. The study met the STROCSS criteria (17).

### Demographics and clinical characteristics

A description of demographic data, such as age and body mass index (BMI), tobacco and alcohol history, comorbidities were obtained from electronic medical record system. Patients were classified into four groups according to BMI: underweight (< 18.5 kg/m^2^), normal weight (18.5 to 24.9 kg/m^2^), overweight (25.0 to 29.9 kg/m^2^), and obese (>30 kg/m^2^) (18). Hypertension, diabetes, and other chronic diseases are considered chronic diseases (e.g., chronic obstructive pulmonary disease). Laboratory data (e.g., serum albumin level, peripheral blood lymphocyte counts, total cholesterol concentration) were measured 3 days before therapy. Organ metastases or bone metastases are diagnosed by an experienced imaging physician using CT, MRI, or bone scans.

### Malnutritional scoring systems

The nutritional status of patients was assessed using three malnutrition scoring systems (NRI, PNI, and CONUT). Calculation formulas were as follows:

NRI: 1.489 ^*^ serum albumin (g/l) + 41.7 ^*^ (weight in kilograms/ideal weight).
PNI: 10 ^*^ serum albumin (g/dl) + 0.005 ^*^ total lymphocyte counts (mm^3^).

CONUT: Scored according to serum albumin level, peripheral blood lymphocyte counts, and total cholesterol concentration (13).

Malnutrition was classified as absent, mild (except PNI), moderate, and severe according to relevant scoring systems (Supplementary Table S1) (13).

### Follow up

All patients with follow-up longer than 6 months were included. Progression-free survival (PFS), PSA-PFS were chosen as the primary endpoints. Progression was defined as a 20% increase in the total diameter of the target lesion as measured by CT scan; the presence of 2 new bone lesions on a bone scan; the progression of symptoms (a deterioration in disease-related symptoms or a new cancer-related complication), or the death of the patient, whichever occurs first. PFS is a composite endpoint (censored) defined as the time to disease progression, onset of symptoms, or death. The traditional approach to analyzing PFS endpoints is based on the time to first observed progression, using a right-censored approach (19). PSA progression is defined as the increase of 25% in PSA from baseline values or an increase of 2 ng/mL or more after 12 weeks (20).

### Cox proportional hazard regression model

In order to identify independent prognostic factors, univariate and multivariate Cox proportional hazard regression models were performed. As the results of the univariable Cox regression analyses were statistically significant (*P* < 0.05), a multivariable Cox proportional hazards regression analysis was carried out. Hazard ratio (HR) was adjusted by age, BMI, chronic diseases, smoking, alcohol, organ metastasis, bone metastasis, castration resistant prostate cancer, Gleason scores, PSA, chemotherapy, surgery, androgen deprivation therapy, and I^125^ seed brachytherapy.

### Subgroup analysis

Postoperative pathological assessment as a high Gleason score (≥8) and a high preoperative PSA level (≥20 ng/ml) were strongly associated with poor prognosis in patients with prostate cancer. In addition, high levels of preoperative PSA can influence the choice of the optimal treatment option (21, 22). Therefore, to further assess the predictive stability of CONUT, NRI, and PNI and to screen which populations are appropriate for each of these three clinical nutritional scoring systems, a subgroup analysis based on pre-operative serum PSA and post-operative pathological Gleason scores was established.

### Bootstrap-based optimism correction

To internally validate the three scoring systems, we performed bootstrap-based optimism correction (23). Briefly, we use the RMS package in R for modeling and use the validata function to compute the C-index correlation results. We focused on the Dxy, Somer's D, which is a transformed version of the c-statistic via Dxy = 2(C-0.5). Then, we calculated the difference in these predictive abilities for each bootstrap sample, and take the average across many bootstrap samples (1,000 times). This estimate of optimism is then subtracted from the naive estimate of predictive power.

### Decision curve analysis

We performed a DCA to assess the non-inferiority of these three clinical nutrition scoring systems compared to the Eighth Edition American Joint Committee on Cancer (AJCC) TNM staging in predicting the prognosis of prostate cancer patients (24). The C-index is used to evaluate the 3-year predictive performance of NRI, PNI, and CONUT alongside the AJCC TNM stage.

### Statistical analysis

Differences in survival between groups were compared using the Kaplan-Meier curve and log-rank tests. PSA-PFS and PFS data were used for Cox proportional hazards regression analysis to assess HR with 95% confidence intervals (CI). Statistically significant results were defined as *P* values < 0.05. For statistical analysis and graphing, IBM SPSS Statistics software (version 26) and R software (version 4.0.3) were used.

## Result

### Patient characteristics

The cohort consisted of 384 male patients with a mean age of 71.35 years. 57.55% of patients had a serum PSA of more than 20 ng/ml at the time of initial diagnosis. Nearly 70 percent had radical robotic-assisted prostatectomy, and 268 patients had androgen deprivation therapy. Table 1 shows the demographic characteristics of the study population.

**Table 1:** Baseline characteristics of the study population.

**Characteristics**	**Overall (*n* = 384)**
**Demographic data**	
Age, years	71.35 (8.10)
BMI, kg/m^2^	24.18 (2.88)
Smoking, yes	41 (10.68%)
Alcohol, yes	61 (15.89%)
**Comorbidities**	
Hypertension	193 (50.26%)
Diabetes	72 (18.75%)
Others	41 (10.68%)
**Disease data**	
**PSA, ng/ml**	
< 20	163 (42.45%)
≥20	221 (57.55%)
**Gleason scores**	
6–7	178 (46.35%)
8–10	206 (53.65%)
CRPC, yes	120 (31.25%)
Organ metastasis^a^, yes	74 (19.27%)
Bone metastasis^a^, yes	194 (50.52%)
ADT, yes	268 (69.80%)
Chemotherapy, yes	19 (4.9%)
Surgery, yes	267 (69.53%)
I^125^ seed brachytherapy	30 (7.81%)
**Nutritional data**	
**NRI**	
Absent	263 (68.49%)
Mild	29 (7.55%)
Moderate	80 (20.83%)
Severe	12 (3.13%)
**PNI**	
Absent	334 (86.97%)
Moderate	29 (7.55%)
Severe	21 (5.49%)
**COUNT**	
Absent	45 (11.72%)
Mild	209 (54.43%)
Moderate	122 (31.77)
Severe	8 (2.08%)

^a^Before or during treatment; Values are mean (SD) or *n* (%); BMI, body mass index; CRPC, castration resistant prostate cancer; ADT, androgen deprivation therapy; CONUT, Controlling Nutritional Status score; PNI, prognostic nutritional index; NRI, nutritional risk index.

### Prevalence of malnutrition

**Figure 1:**
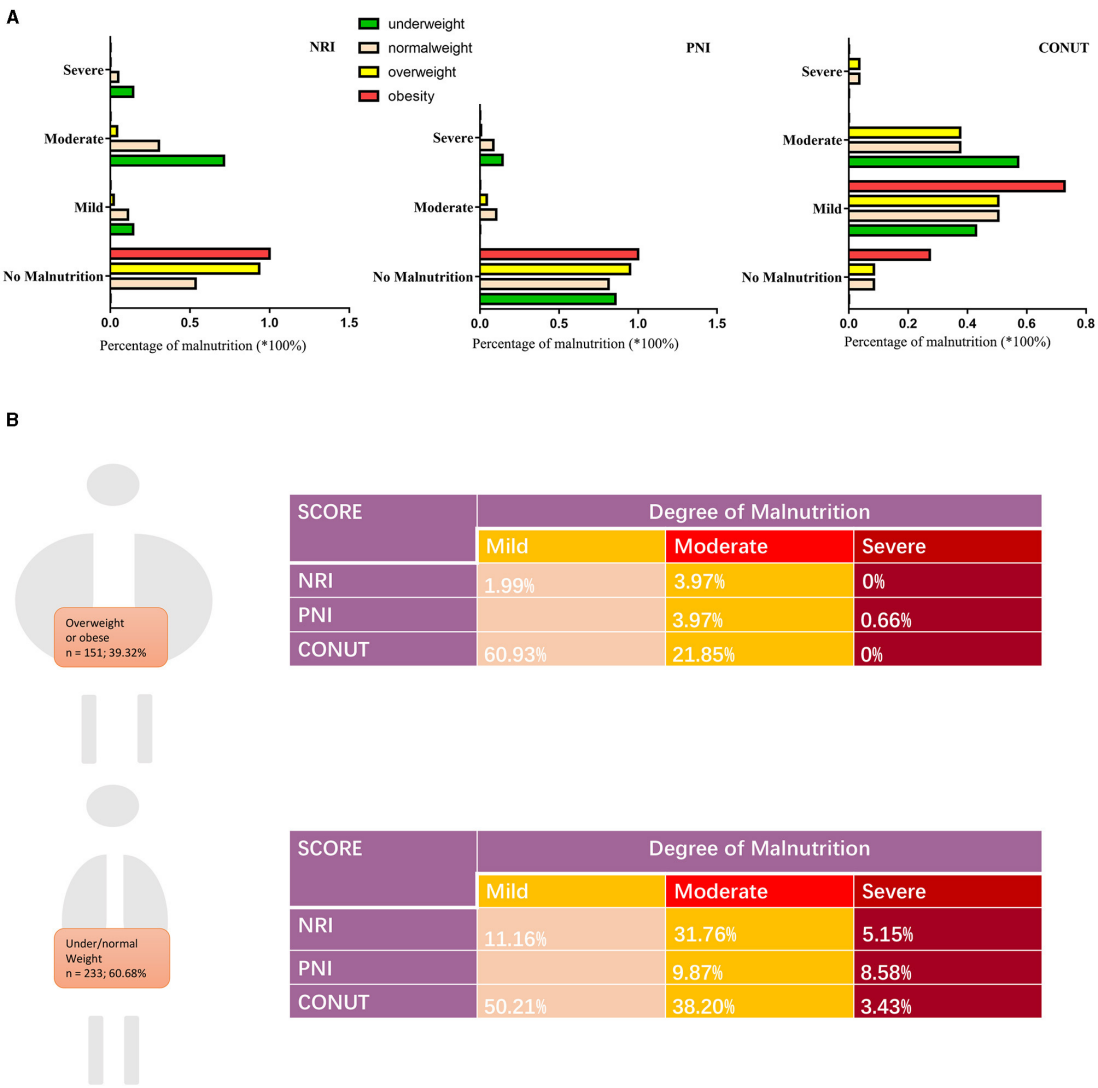
Prevalence of malnutrition by BMI in this cohort. **(A)** Percentage of malnutrition by three scoring systems according to BMI: underweight (< 18.5 kg/m^2^), normal weight (18.5 to 24.9 kg/m^2^), overweight (25.0 to 29.9 kg/m^2^), and obese (>30 kg/m^2^). **(B)** Degree of malnutrition by three scoring systems according to body weight. BMI, body mass index; NRI, nutritional risk index; PNI, prognostic nutritional index; CONUT, Controlling Nutritional Status score.

Using BMI, we classified the patients into four groups (underweight, normal weight, overweight, and obese) and the prevalence of malnutrition in different scoring systems were displayed accordingly (Figure 1). A total of 151 (39.32%) patients were classified into overweight/obesity. There were 233 (60.68%) patients belongs to low/normal weight.

### Association of the three scoring systems with PSA-PFS and PFS

**Figure 2:**
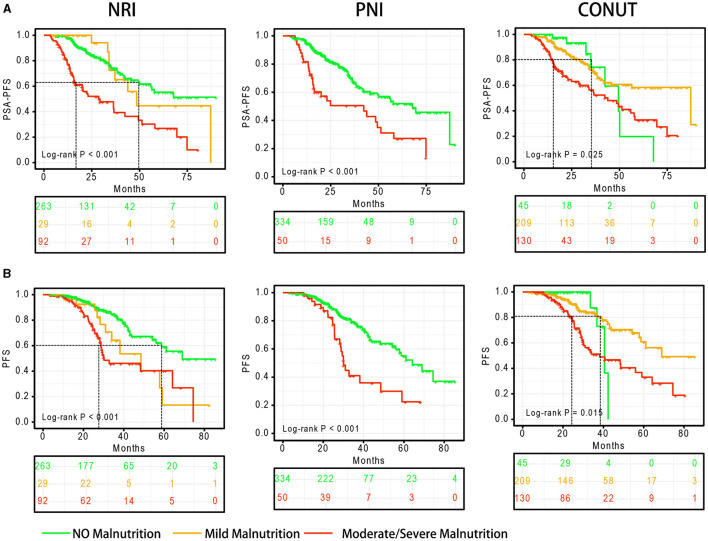
Survival curves based on the degree of malnutrition. **(A)** PSA-PFS, **(B)** PFS. PFS, progress free survival; NRI, nutritional risk index; PNI, prognostic nutritional index; CONUT, Controlling Nutritional Status score.

Malnutrition status is strongly associated with poor PSA-PFS (Figure 2A). In the NRI scoring system, the median PSA-PFS for mild and moderate/severe malnutrition was 48.5 (log-rank *P* = 0.813) and 28.7 months (log-rank *P* < 0.001), respectively. In the PNI scoring system, the median PSA-PFS for normal nutritional status and moderate/severe malnutrition was 67.8 and 42.3 months (log-rank *P* < 0.001), respectively. Moreover, In the CONUT scoring system, the median PSA-PFS for moderate/severe malnutrition was 43.9 months (log-rank *P* = 0.025).

Malnutrition status is also associated with of poor PFS (Figure 2B). In the NRI scoring system, the median PFS for mild and moderate/severe malnutrition was 48.5 (log-rank *P* = 0.012) and 30.2 months (log-rank *P* < 0.001), respectively. In the PNI scoring system, the median PFS for normal nutritional status and moderate/severe malnutrition was 64.2 and 30.1 months (log-rank *P* < 0.001), respectively. Moreover, In the CONUT scoring system, the median PFS for moderate/severe malnutrition was 38.1 months (log-rank *P* = 0.015).

**Figure 3:**
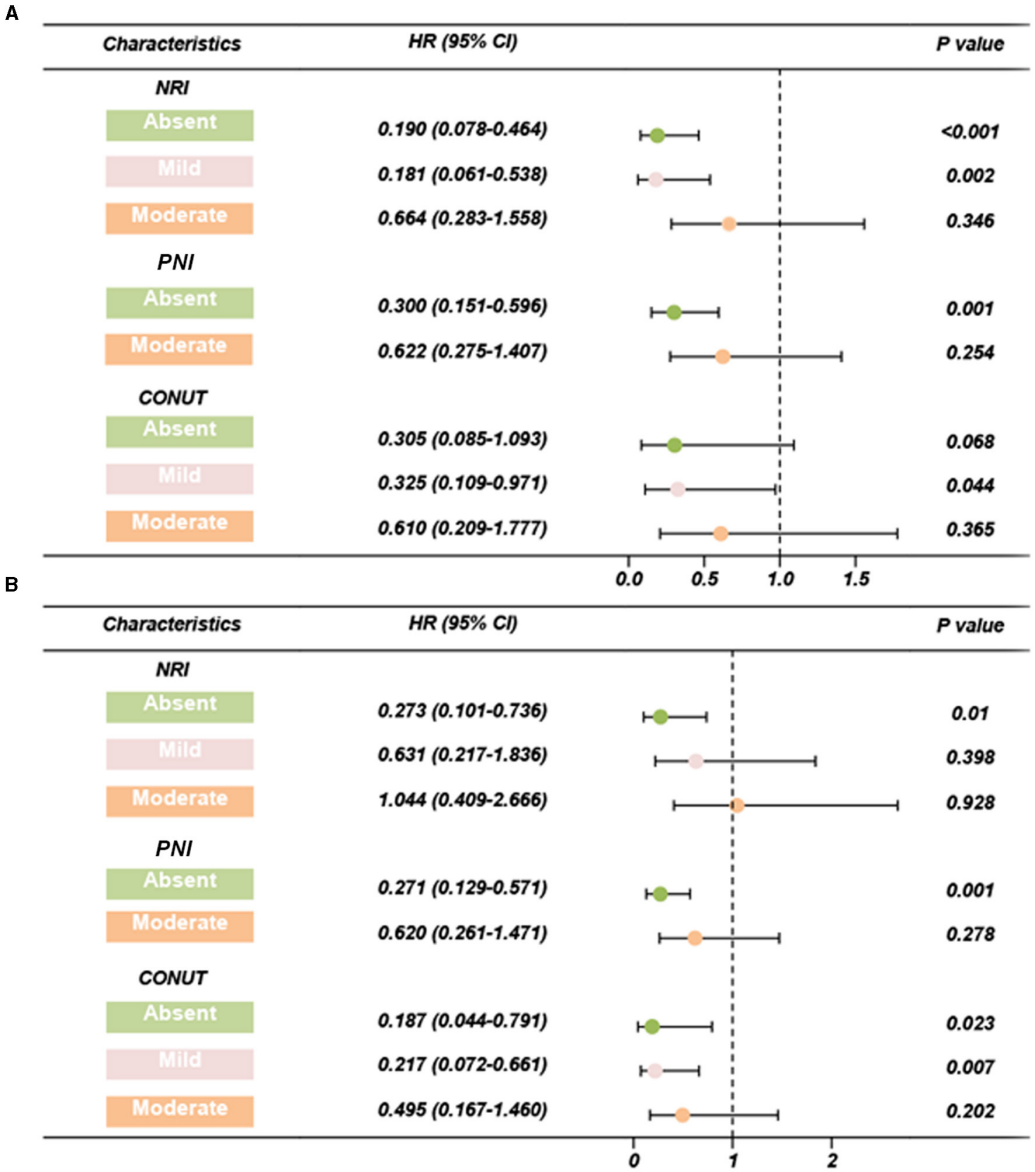
Forest plots of adjusted HR based on three scoring systems (adjusted by age, BMI, chronic diseases, smoking, alcohol, organ metastasis, bone metastasis, castration resistant prostate cancer, Gleason scores, PSA, chemotherapy, surgery, androgen deprivation therapy, I^125^ seed brachytherapy). **(A)** PSA-PFS, **(B)** PFS. PFS, progress free survival; NRI, nutritional risk index; PNI, prognostic nutritional index; CONUT, Controlling Nutritional Status score; HR, hazard ratio; CI, confidence interval.

Cox regression analyses of the three scoring systems for predicting PSA-PFS and PFS are presented in Tables 2, 3. The forest plots of the adjusted HR based on the three scoring systems are shown in Figure 3. The results of multivariable Cox regression analyses showed that normal nutritional status as defined by NRI (HR = 0.190, 95%CI 0.078, 0.464; *P* < 0.001) and PNI (HR = 0.300 95%CI 0.151, 0.596; *P* = 0.001) is an independent protective factor for PSA-PFS. However, the nutritional status as assessed by CONUT is spurious to predict PSA-PFS. Normal nutritional status as assessed by NRI (HR = 0.273, 95%CI 0.101, 0.736; *P* = 0.010), PNI (HR = 0.271, 95%CI 0.129, 0.571; *P* = 0.001), and CONUT (HR = 0.187, 95%CI 0.044, 0.791; *P* = 0.023) systems was an independent prognostic protective factor for PFS.

**Table 2:** Cox proportional hazards regression analyses of malnutrition indexes to predict PSA-PFS for patients with PCa.

**Characteristics**	**Crude HR (95% CI)**	***P* value**	**Adjusted HR (95% CI) ^a^**	***P* value**	**Adjusted HR (95% CI) ^b^**	***P* value**
**NRI**						
Severe	Reference		Reference		Reference	
Moderate	0.581 (0.259–1.304)	0.188				
Mild	0.200 (0.071–0.561)	0.002	0.193 (0.066–0.570)	0.003	0.181 (0.061–0.538)	0.002
Absent	0.179 (0.081–0.395)	< 0.001	0.207 (0.085–0.501)	< 0.001	0.190 (0.078–0.464)	< 0.001
**PNI**						
Severe	Reference		Reference		Reference	
Moderate	0.642 (0.302–1.365)	0.250				
Absent	0.310 (0.168–0.570)	< 0.001	0.358 (0.186–0.691)	0.002	0.300 (0.151–0.596)	0.001
**COUNT**						
Severe	Reference		Reference		Reference	
Moderate	0.643 (0.231–1.787)	0.397				
Mild	0.301 (0.108–0.838)	0.022	0.381 (0.132–1.097)	0.074		
Absent	0.273 (0.082–0.912)	0.035	0.353 (0.101–1.230)	0.102		

^a^Adjusted by age, BMI, chronic diseases, smoking, alcohol, organ metastasis, bone metastasis, castration resistant prostate cancer, Gleason scores, PSA. ^b^Adjusted by age, BMI, chronic diseases, smoking, alcohol, organ metastasis, bone metastasis, castration resistant prostate cancer, Gleason scores, PSA, chemotherapy, surgery, androgen deprivation therapy, I^125^ seed brachytherapy. HR, hazard ratio; CI, confidence interval; CONUT, Controlling Nutritional Status score; PNI, prognostic nutritional index; NRI, nutritional risk index.

**Table 3:** Cox proportional hazards regression analyses of malnutrition indexes to predict PFS for patients with PCa.

**Characteristics**	**Crude HR (95% CI)**	***P* value**	**Adjusted HR (95% CI) ^a^**	***P* value**	**Adjusted HR (95% CI) ^b^**	***P* value**
**NRI**						
Severe	Reference		Reference		Reference	
Moderate	0.664 (0.274–1.611)	0.365				
Mild	0.509 (0.186–1.393)	0.188				
Absent	0.230 (0.096–0.549)	0.001	0.224 (0.084–0.602)	0.003	0.273 (0.101–0.736)	0.010
**PNI**						
Severe	Reference		Reference		Reference	
Moderate	0.632 (0.278–1.435)	0.272				
Absent	0.271 (0.137–0.539)	< 0.001	0.300 (0.145–0.621)	0.001	0.271 (0.129–0.571)	0.001
**COUNT**						
Severe	Reference		Reference		Reference	
Moderate	0.599 (0.213–1.680)	0.330				
Mild	0.236 (0.083–0.671)	0.007	0.259 (0.087–0.770)	0.015	0.217 (0.072–0.661)	0.007
Absent	0.196 (0.049–0.785)	0.021	0.225 (0.054–0.939)	0.041	0.187 (0.044–0.791)	0.023

^a^Adjusted by age, BMI, chronic diseases, smoking, alcohol, organ metastasis, bone metastasis, castration resistant prostate cancer, Gleason scores, PSA. ^b^Adjusted by age, BMI, chronic diseases, smoking, alcohol, organ metastasis, bone metastasis, castration resistant prostate cancer, Gleason scores, PSA, chemotherapy, surgery, androgen deprivation therapy, I^125^ seed brachytherapy. HR, hazard ratio; CI, confidence interval; CONUT, Controlling Nutritional Status score; PNI, prognostic nutritional index; NRI, nutritional risk index.

Subgroup analysis showed that neither pre-operative serum PSA levels nor post-operative pathological Gleason scores affected NRI's prognostic performance on PSA-PFS and PFS, suggesting that it may be appropriate for all PCa patients to undergo clinical nutrition assessment. In addition, PNI also performed well except for Gleason level (Gleason score ≥ 8) on PSA-PFS. Overall, the results of CONUT's subgroup analysis based on both PSA levels and Gleason scores were unstable, with poor predictive power for patients with high PSA levels (PSA ≥ 20 ng/ml) and Gleason scores (Gleason score ≥ 8) on both PSA-PFS and PFS (Tables 4, 5). This means that the clinical application of CONUT is limited and can only be used in selected patients.

**Table 4:** Subgroup analysis of malnutrition status and PSA-PFS in patients with prostate cancer.

**Characteristic**		**HR (95% CI)**	***P* value**	**HR (95% CI)**	***P* value**	**HR (95% CI)**	***P* value**	
**NRI**	**Severe**	**Moderate**		**Mild**		**Absent**		
Overall	Reference	0.581 (0.259–1.304)	0.188	0.200 (0.071–0.561)	0.002	0.179 (0.081–0.395)	< 0.001	
PSA < 20 ng/ml	Reference	0.228 (0.047–1.107)	0.067	0.114 (0.020–0.641)	0.014	0.088 (0.019–0.400)	0.002	
PSA ≥ 20 ng/ml	Reference	0.738 (0.284–1.918)	0.533	0.147 (0.028–0.759)	0.022	0.222 (0.086–0.573)	0.002	
Gleason score < 8	Reference	0.224 (0.060–0.833)	0.026	0.077 (0.016–0.364)	0.001	0.051 (0.014–0.187)	< 0.001	
Gleason score ≥ 8	Reference	0.809 (0.278–2.352)	0.697	0.236 (0.052–1.062)	0.060	0.318 (0.112–0.904)	0.032	
**PNI**	**Severe**	**Moderate**		**Absent**				
Overall	Reference	0.642 (0.302–1.365)	0.250	0.310 (0.168–0.570)	< 0.001			
PSA < 20 ng/ml	Reference	0.190 (0.046–0.789)	0.022	0.078 (0.022–0.284)	< 0.001			
PSA ≥ 20 ng/ml	Reference	0.807 (0.300–2.175)	0.672	0.432 (0.212–0.879)	0.021			
Gleason score < 8	Reference	0.129 (0.034–0.483)	0.002	0.086 (0.030–0.248)	< 0.001			
Gleason score ≥ 8	Reference	1.093 (0.414–2.885)	0.857	0.473 (0.213–1.052)	0.066			
**COUNT**	**Severe**	**Moderate**		**Mild**		**Absent**		
Overall	Reference	0.643 (0.231–1.787)	0.397	0.301 (0.108–0.838)	0.022	0.273 (0.082–0.912)	0.035	
PSA < 20 ng/ml	Reference	0.224 (0.028–1.779)	0.157	0.119 (0.015–0.938)	0.043	0.095 (0.008–1.071)	0.057	
PSA ≥ 20 ng/ml	Reference	0.833 (0.254–2.732)	0.763	0.386 (0.117–1.273)	0.118	0.354 (0.088–1.425)	0.144	
Gleason score < 8	Reference	0.215 (0.048–0.973)	0.046	0.076 (0.017–0.353)	0.001	0.084 (0.015–0.481)	0.005	
Gleason score ≥ 8	Reference	0.974 (0.231–4.114)	0.971	0.571 (0.136–2.394)	0.443	0.480 (0.088–2.630)	0.398	

CONUT, Controlling Nutritional Status score; PNI, prognostic nutritional index; NRI, nutritional risk index; HR, hazard ratio; CI, confidence interval.

**Table 5:** Subgroup analysis of malnutrition status and PFS in patients with prostate cancer.

**Characteristic**		**HR (95% CI)**	***P* value**	**HR (95% CI)**	***P* value**	**HR (95% CI)**	***P* value**	
**NRI**	**Severe**	**Moderate**		**Mild**		**Absent**		
Overall	Reference	0.664 (0.274–1.611)	0.365	0.509 (0.186–1.393)	0.188	0.230 (0.096–0.549)	0.001	
PSA < 20 ng/ml	Reference	0.168 (0.031–0.908)	0.038	0.458 (0.094–2.239)	0.335	0.115 (0.025–0.525)	0.005	
PSA ≥ 20 ng/ml	Reference	1.118 (0.386–3.234)	0.838	0.419 (0.093–1.886)	0.257	0.317 (0.109–0.921)	0.035	
Gleason score < 8	Reference	0.658 (0.146–2.968)	0.586	0.476 (0.092–2.450)	0.375	0.136 (0.030–0.614)	0.009	
Gleason score ≥ 8	Reference	0.596 (0.197–1.806)	0.360	0.547 (0.146–2.050)	0.370	0.327 (0.112–0.952)	0.040	
**PNI**	**Severe**	**Moderate**		**Absent**				
Overall	Reference	0.632 (0.278–1.435)	0.272	0.271 (0.137–0.539)	< 0.001			
PSA < 20 ng/ml	Reference	0.371 (0.092–1.490)	0.162	0.097 (0.027–0.351)	< 0.001			
PSA ≥ 20 ng/ml	Reference	0.785 (0.271–2.277)	0.656	0.430 (0.190–0.973)	0.043			
Gleason score < 8	Reference	0.436 (0.116–1.644)	0.220	0.185 (0.062–0.550)	0.002			
Gleason score ≥ 8	Reference	0.799 (0.280–2.276)	0.674	0.342 (0.140–0.832)	0.018			
**COUNT**	**Severe**	**Moderate**		**Mild**		**Absent**		
Overall	Reference	0.599 (0.213–1.680)	0.330	0.236 (0.083–0.671)	0.007	0.196 (0.049–0.785)	0.021	
PSA < 20 ng/ml	Reference	0.188 (0.040–0.870)	0.033	0.059 (0.012–0.285)	< 0.001	0.058 (0.005–0.649)	0.021	
PSA ≥ 20 ng/ml	Reference	1.203 (0.285–5.070)	0.802	0.537 (0.126–2.287)	0.400	0.384 (0.064–2.302)	0.295	
Gleason score < 8	Reference	0.416 (0.096–1.806)	0.241	0.084 (0.018–0.393)	0.002	0.108 (0.015–0.775)	0.027	
Gleason score ≥ 8	Reference	0.730 (0.170–3.136)	0.672	0.456 (0.107–1.946)	0.289	0.320 (0.045–2.277)	0.255	

CONUT, Controlling Nutritional Status score; PNI, prognostic nutritional index; NRI, nutritional risk index; HR, hazard ratio; CI, confidence interval.

**Figure 4:**
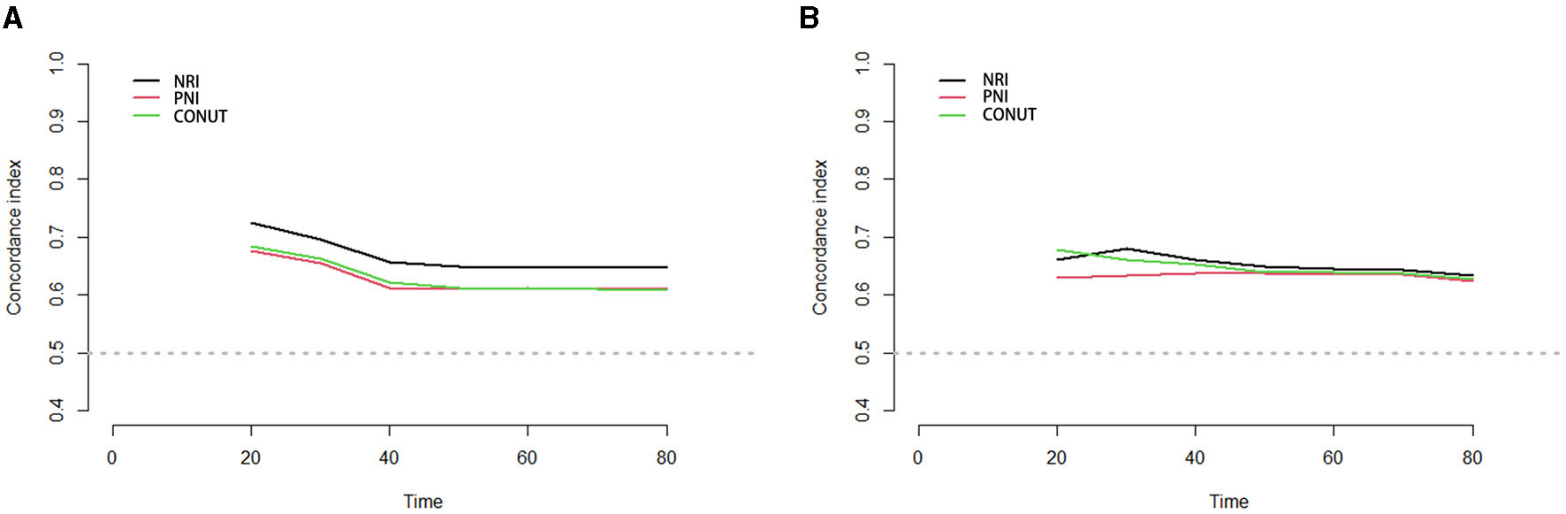
Time-C-Index plot based on three scoring systems (adjusted by age, BMI, chronic diseases, smoking, alcohol, organ metastasis, bone metastasis, castration resistant prostate cancer, Gleason scores, PSA, chemotherapy, surgery, androgen deprivation therapy, I^125^ seed brachytherapy). **(A)** PSA-PFS, **(B)** PFS. Time, months; PFS, progress free survival; NRI, nutritional risk index; PNI, prognostic nutritional index; CONUT, Controlling Nutritional Status score.

Internal validation and optimism correction approaches were performed to validate the three scoring systems. In Figure 4, the adjusted C-index is plotted for different scoring systems and different time points to compare the prediction performance. When predicting PSA-PFS, the C-index decreases over time for all three scoring systems. However, the prediction performance stabilizes after 40 months. The NRI had the highest C-index (0.731), and the optimistic estimate of the corrected C-index was 0.703. When predicting the PFS, the C-index of the NRI is still the highest (0.726), and the optimistic estimate of the corrected c-index is 0.692 (Table 6).

**Table 6:** Bootstrap-based optimism correction of adjusted C-index^a^.

	**C-index**	**C-index after optimistic estimate correction**
**PSA-PFS**		
NRI	0.731	0.703
PNI	0.694	0.661
CONUT	0.698	0.666
**PFS**		
NRI	0.726	0.692
PNI	0.703	0.665
CONUT	0.719	0.683

CONUT, Controlling Nutritional Status score; PNI, prognostic nutritional index; NRI, nutritional risk index. ^a^Adjusted by age, BMI, chronic diseases, smoking, alcohol, organ metastasis, bone metastasis, castration resistant prostate cancer, Gleason scores, PSA, chemotherapy, surgery, androgen deprivation therapy, I^125^ seed brachytherapy.

**Figure 5:**
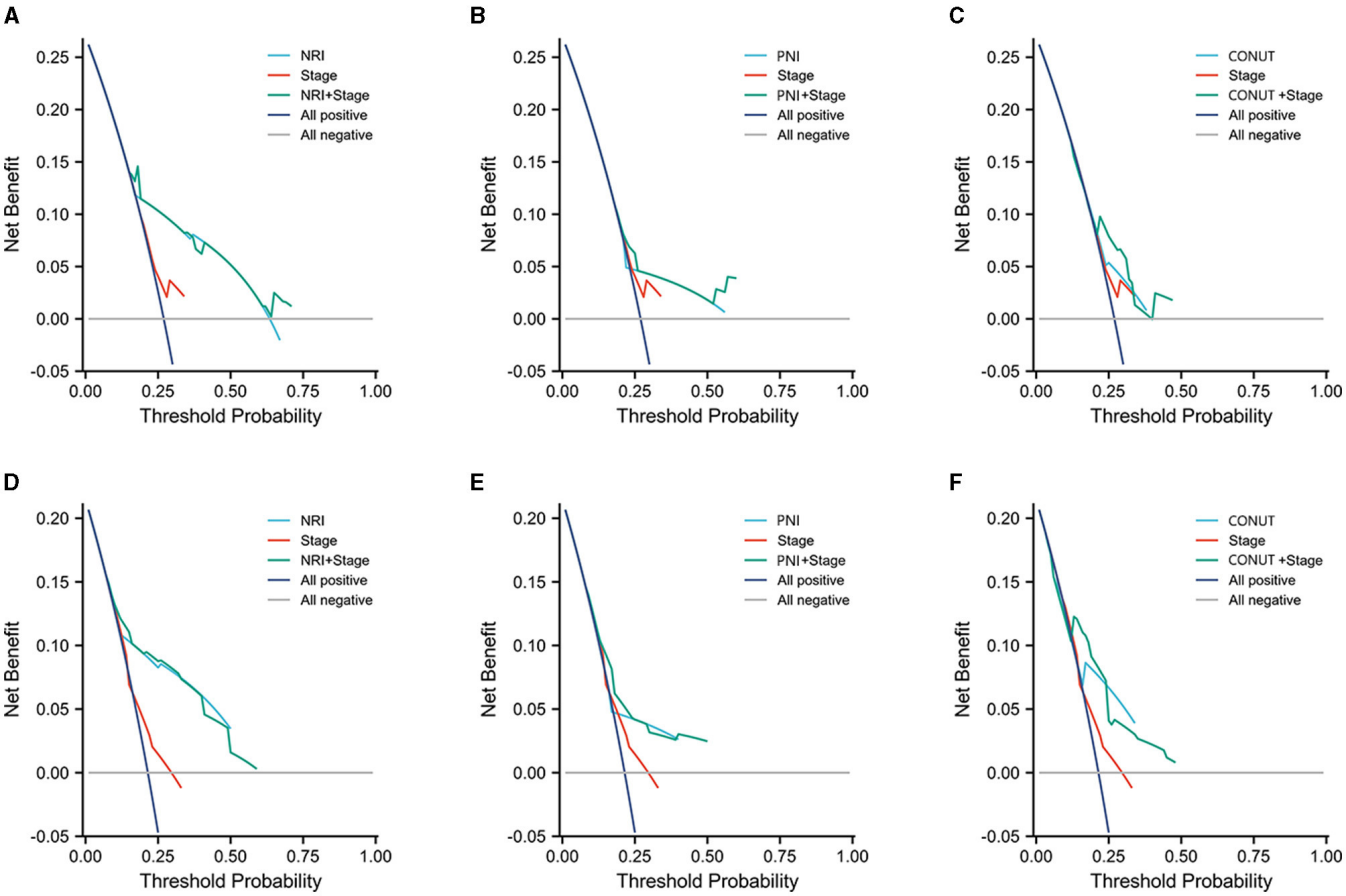
Three years decision curve analysis (DCA) on performance of three scoring systems and AJCC stage. **(A–C)** PSA-PFS, **(D–F)** PFS.

To enable the comparison of various predictive models, DCA is employed. Figure 5 depicts the clinical utility of each model across a range of potential thresholds for PFS and PSA-PFS on the x-axis, and the net benefit of utilizing the model for patient risk stratification on the y-axis, relative to the assumption that no patient will experience progression. In this analysis, the AJCC curve closely approximates the two extreme curves (all positive, all negative), indicating minimal clinical utility. In contrast, the NRI, PNI, and CONUT all demonstrate superior benefits compared to the extreme curves across a broad spectrum of thresholds. Consequently, they offer a relatively extensive and secure range of thresholds for selection. Notably, NRI surpasses both PNI and CONUT in predicting PFS and PSA-PFS. Furthermore, the C-index for all three scoring systems, as presented in Table 7, exceeds that of the AJCC stage. The integration of the AJCC stage with the nutritional scoring systems provides a more accurate prediction of PSA-PFS and PFS in prostate cancer patients than the AJCC stage alone. For PSA-PFS, NRI has the highest C-index (0.737, 95%CI 0.701–0.774), followed by CONUT (0.635, 95%CI 0.597–0.673), and then PNI (0.634, 95%CI 0.598–0.671). For PFS, NRI also showed the highest C-index (0.750, 95%CI 0.713–0.788), followed by CONUT (0.675, 95%CI 0.637–0.714), and then PNI (0.638, 95%CI 0.599–0.677).

**Table 7:** Model performance of each scoring system for PSA-PFS and PFS.

**Characteristics**	**PSA-PFS**	**PFS**
	**C-index**	**C-index**
NRI	0.737 (0.701–0.774)	0.750 (0.713–0.788)
PNI	0.634 (0.598–0.671)	0.638 (0.599–0.677)
CONUT	0.635 (0.597–0.673)	0.675 (0.637–0.714)
Model	C-index	C-index
TNM stage	0.578 (0.533–0.622)	0.609 (0.567–0.652)
TNM + NRI	0.764 (0.728–0.799)	0.787 (0.753–0.821)
TNM + PNI	0.679 (0.636–0.722)	0.717 (0.678–0.756)
TNM + CONUT	0.670 (0.632–0.709)	0.725 (0.690–0.760)

CONUT, Controlling Nutritional Status score; PNI, prognostic nutritional index; NRI, nutritional risk index.

## Discussion

Patients often suffer from malnutrition, which is a common public health problem worldwide (25). The implementation of non-invasive strategies for the accurate prediction of tumor prognosis in PCa patients remains an unmet clinical need. This study aimed to evaluate the prognostic significance of three nutritional scoring systems within a cohort of PCa patients. Notably, more than half of the patients in this cohort presented with comorbid chronic diseases, such as hypertension, diabetes mellitus, and respiratory disorders. These chronic conditions contribute to diminished nutritional intake and increased muscle wasting, which are further aggravated by systemic inflammation-a primary factor in the development of malnutrition (26, 27). Similarly, PSA level, Gleason score, treatment, and metastatic status are risk factors for postoperative biochemical recurrence and PFS in PCa patients (28–30). Consequently, we excluded these confounding variables during the multivariate Cox regression analyses and determined that normal nutritional status, as indicated by NRI and PNI scores, served as an independent protective factor for PSA-PFS. To further elucidate the predictive stability of each of the three nutritional scoring systems in relation to PSA levels and Gleason scores, we conducted subgroup analyses. The findings revealed that neither PSA levels nor Gleason scores influenced the prognostic performance of the NRI on PSA-PFS and PFS.

Previous studies have revealed that cancer-related malnutrition leads to poorer hospital outcomes and survival and increases the cost of care (31–33). According to recent ESPEN recommendations, each cancer patient should undergo a multidimensional clinical nutritional assessment. Individualized treatment plans to increase nutritional intake, reduce inflammation, and increase physical activity from early in the treatment process (34). Two clinical phase III, multicenter, double-blind, randomized controlled trials (COU-AA-301 and COU-AA-302) uncovered the relationship between prostate cancer-related malnutrition, and survival (35, 36). Researchers found that low albumin was an independent risk factor for poor overall survival (OS), while low BMI did not. It is more accurate to classify risk groups based on albumin and BMI combined (37). High BMI (>25 kg/m^2^) is associated with longer OS and lower prostate cancer-specific mortality in several studies (38, 39). However, in the AX327 clinical trial, there was no significant correlation between BMI and OS (40). Previous studies had explained the relationship between BMI and survival under different treatment strategies (next-generation anti-androgen treatment or docetaxel-based chemotherapy). Adipose tissue is known to convert androgens into estrogens, which improves survival by circulating estradiol (41). Due to its lipophilic nature, docetaxel had a higher volume of distribution and less distal efficacy in patients with higher body mass indexes (42). As a result, a single BMI does not truly reflect the nutritional status of patients under different treatment strategies and may not be an applicable predictor of survival in PCa patients. In this cohort, NRI demonstrated the best predictive value. Serum albumin is a convenient biochemical indicator of nutritional and inflammatory status. It can identify patients who are actually cachectic from those with high BMI (37). The NRI scoring system integrates serum albumin with anthropometric factors that can improve the diagnostic value.

The CONUT score is derived from serum albumin concentration, peripheral lymphocyte counts, and total cholesterol concentration, providing a comprehensive evaluation of synthetic metabolism and host immune function. Previous studies have identified a poor preoperative CONUT score as an independent risk factor for poor prognosis in patients with localized upper tract urothelial carcinoma (43) and bladder cancer (44) undergoing radical surgery. Furthermore, a high CONUT score has been significantly associated with an increased incidence of major perioperative complications in patients undergoing radical cystectomy (44). A meta-analysis has highlighted a significant correlation between reduced total cholesterol (TC) levels and diminished survival rates in various tumor types (45). The potential biological mechanisms underlying this association are as follows: firstly, serum cholesterol levels may influence intracellular signaling pathways, thereby diminishing the effectiveness of antitumor therapies (46); secondly, serum TC is indicative of the body's energy reserves, with decreased TC levels reflecting caloric depletion within tumor tissues (47); and thirdly, lower serum TC levels may result in increased concentrations of cancer-related proteins, such as elevated serum interleukin-6 levels (48). However, other studies do not corroborate the role of TC as a risk factor for the severity or prognosis of PCa following prostatectomy (49, 50). The findings from the REDUCE study indicate that elevated TC is associated with an increased risk of high-grade PCa, potentially attributable to the aberrant cholesterol metabolism in prostate cancer cells and the synthesis of androgens that facilitate tumor cell growth (51). In the context of the CONUT score, a high cholesterol level is considered an indicator of good nutritional status, which contrasts with the abnormal cholesterol metabolism observed in PCa. In our subgroup analysis, the performance of the CONUT score was found to be unstable, failing to predict elevated PSA levels and the prognosis of Gleason score subgroups. This instability may be attributed, at least in part, to the inclusion of TC in the CONUT score. Serum albumin and peripheral blood lymphocytes, which are components of the PNI, are indicative of systemic inflammation and immune status. Consequently, the PNI demonstrates an advantage in predicting postoperative complications. Supporting this, studies by Yu et al. (52) and Wang et al. (53) have reported that patients with low preoperative PNI scores experienced higher rates of complications following radical cystectomy. Although a lower peripheral blood lymphocyte count has been reported to be associated with poor prognosis in prostate cancer (54), the tumor microenvironment (TME) in primary and castration-resistant prostate cancer exhibits a relative lack of immune infiltration compared to other malignant tumors (55). Consequently, the predictive value of peripheral blood lymphocyte counts for the prognosis of prostate cancer patients warrants further investigation.

Historically, hospitalized patients undergoing radical prostatectomy were subjected to pelvic lymph node dissection (PLND) to achieve precise pathological staging. This procedure, however, significantly elevated the risk of postoperative complications and posed substantial challenges for malnourished patients (56). Currently, advancements in technology, such as Prostate-Specific Membrane Antigen Positron Emission Tomography (PSMA-PET), have enhanced the accuracy of oncological assessments and mitigated the surgical complications associated with malnutrition risk (57, 58). This study examines the prognostic utility of three nutritional scoring systems in patients with prostate cancer (PCa) and aims to inform clinical nutritional interventions. However, there is a paucity of research on preoperative nutritional support for prostate cancer patients. Future investigations that extend preoperative nutritional assessments and explore the clinical implications of nutritional interventions in this patient population would be highly valuable.

This study has the following limitations: First, the number of cases in this cohort is insufficient. Second, this is a retrospective study, which is inherently flawed. In the future, further validation of the role of the three scoring systems in predicting survival of prostate cancer is needed in prospective multicenter studies with large samples.

In this study, we used NRI, PNI, and CONUT to evaluate the nutritional status of prostate cancer patients and its link to prognosis. Through various analyses, we identified independent prognostic factors and suitable populations for each scoring system. Our findings indicate that NRI offers superior predictive stability, broader applicability, and better performance.

## Data Availability

The raw data supporting the conclusions of this article will be made available by the authors, without undue reservation.
