# Supplementary material for: Prognostic value of three clinical nutrition scoring system (NRI, PNI, and CONUT) in elderly patients with prostate cancer
Source: Front Nutr. 2024 Oct 1;11:1436063. doi: 10.3389/fnut.2024.1436063 (PMC11473420; doi:10.3389/fnut.2024.1436063)
Supplement: Supplementary file 1 [file Table_1.DOCX]

| **Nutritional indices** | **Risk of Malnutrition** | | | |
| --- | --- | --- | --- | --- |
|  | **Absent** | **Mild** | **Moderate** | **Severe** |
| **CONUT, points** | 0-1 | 2-4 | 5-8 | 9-12 |
| Albumin, g/dl (score)  Total cholesterol, mg/dl (score)  Lymphocyte count, *10^9^/l (score) | ≥3.5 (0) | 3.0-3.4 (2) | 2.5-2.9 (4) | <2.5 (6) |
|  | ≥180 (0) | 140-199 (1) | 100-139 (2) | <100 (3) |
|  | ≥1.60 (0) | 1.20-1.59 (1) | 0.80-1.19 (2) | <0.80 (3) |
| **NRI, points** | ≥100 | 97.50-99.99 | 83.50-97.49 | <83.50 |
| **PNI, points** | >38 | - | 35-38 | <35 |

Table S1. Procedures for the evaluation of each nutritional index
